# Correction: No evidence for parallel evolution of cursorial limb adaptations among Neogene South American native ungulates (SANUs)

**DOI:** 10.1371/journal.pone.0331100

**Published:** 2025-08-26

**Authors:** Darin A. Croft, Malena Lorente

The captions of Figs 1 to 3 were incorrectly captured as text. Please see the correct Figs 1 to 3 here.

**Fig 1:**
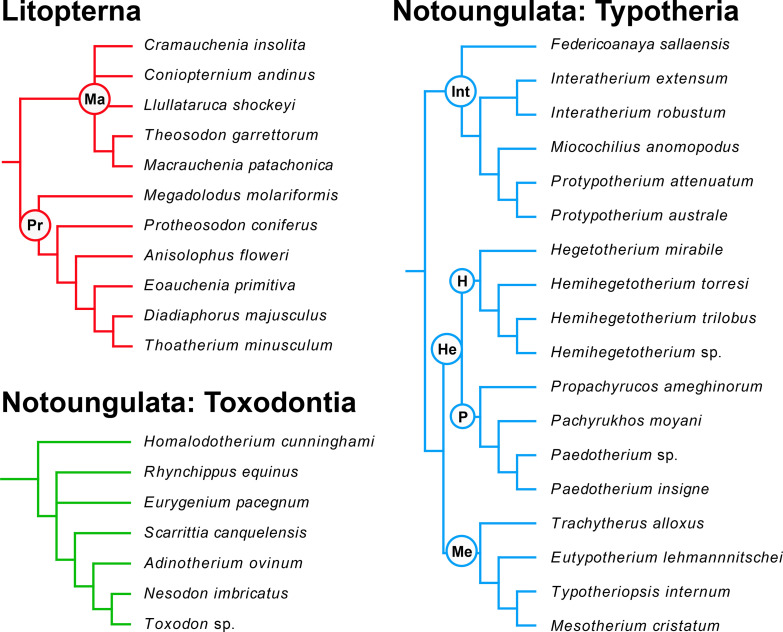
Phylogenetic relationships of SANUs analyzed in this study, grouped by major clade. Litoptern relationships are based on McGrath et al. [60, 61], with families Macraucheniidae (Ma) and Proterotheriidae (Pr) indicated. Toxodontian relationships are based on Shockey et al. [62], Bonini et al. [63], and Armella et al. [64]. Typothere relationships are based on Seoane and Cerdeño [65] and Croft and Anaya [66], with *Hemihegetotherium* sp. assumed to be closely related to *Hemihegetotherium achataleptum*. Typothere clades include Hegetotheriidae (He), Hegetotheriinae **(H)**, Interatheriidae (Int), Mesotheriidae (Me), and Pachyrukhinae **(P)**.

**Fig 2:**
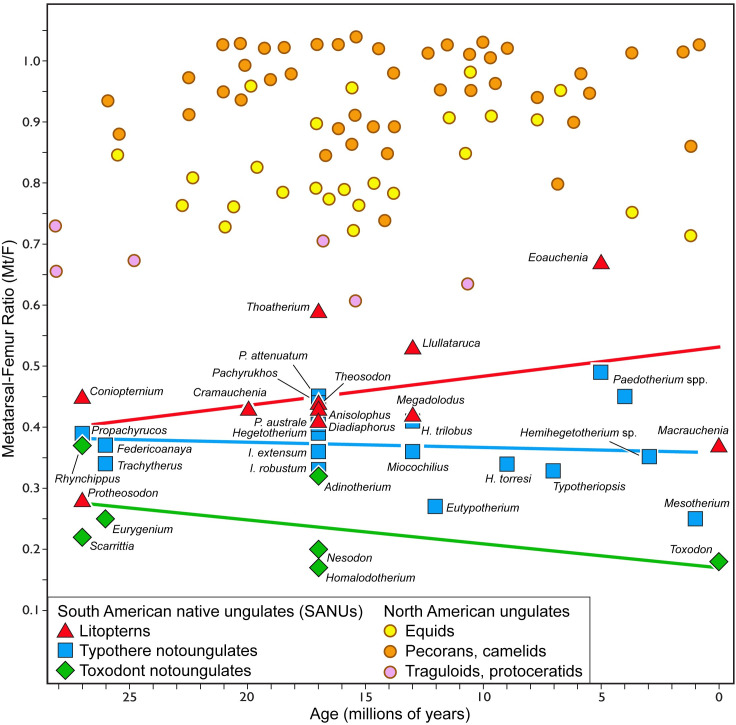
Metatarsal-femur ratio (Mt:F) in extinct North American ungulates and South American native ungulates. North American ungulate data (N = 73) are from Janis and Wilhelm [28]. Data for South American native ungulates (N = 36) are provided in S1 and S2 Tables. Regression lines are ordinary least-squared regressions.

**Fig 3:**
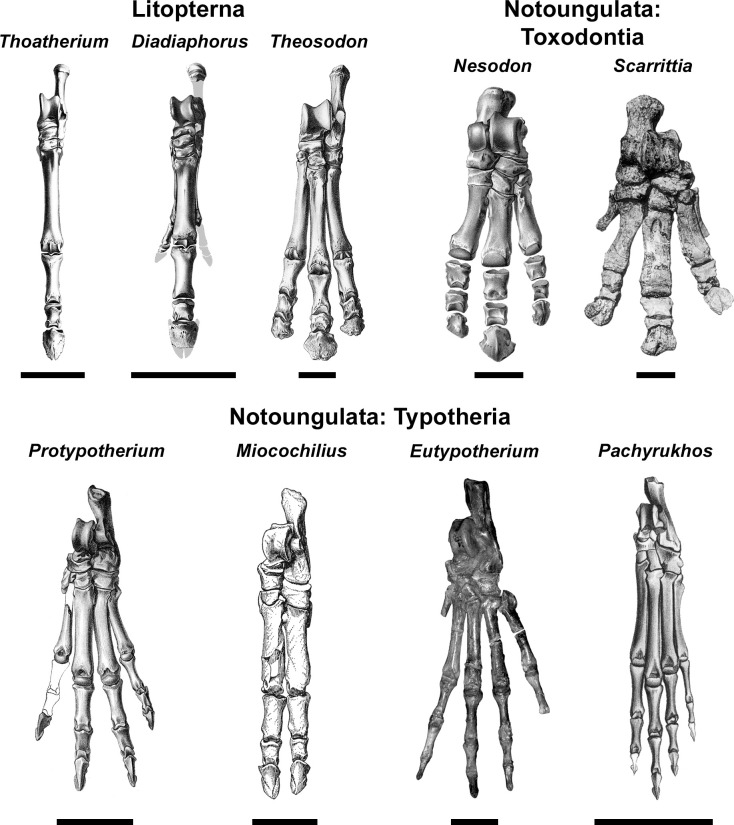
The pes of representative South American native ungulates. Taxa include: *Thoatherium minusculum* (Proterotheriidae), YPPM-VPPU 15719 (reversed; from Scott 1910, pl. XIII, fig. 13 [78]); *Diadiaphorus majusculus* (Proterotheriidae), AMNH 9196 (modified from Scott 1910, pl. V, fig. 2 [78]); *Theosodon lydekkeri* (Macraucheniidae), AMNH 9269 (from Scott 1910, pl. XX, fig. 7 [78]); *Nesodon imbricatus* (Toxodontidae), YPPM-VPPU 15968, (from Scott 1912, pl. XXV, fig. 9 [79]); *Scarrittia canquelensis* (Leontiniidae), AMNH 29585 (from Chaffee 1952, pl. 11, fig. 2 [80]); *Protypotherium australe* (Interatheriidae), AMNH 9149 (from Sinclair 1909, pl. V, fig. 1 [81]); *Miocochilius anomopodus* (Interatheriidae), UCMP 38091 (from Stirton 1953, pl. 27 [75]); *Eutypotherium lehmannnitchei* (Mesotheriidae), MLP 12-1701 (reversed); *Pachyrukhos moyani* (Hegetotheriidae), AMNH 9481 (reversed; from Sinclair 1909, pl. X, fig. 15 [81]). Scale bars equal 5 cm in upper row (litopterns, toxodonts) and 3 cm in lower row (typotheres).

As result of the correction to the Figure captions, the following information should not be included in the Introduction, Results and Discussion respectively.

Litoptern relationships are based on McGrath et al. [60, 61], with families Macraucheniidae (Ma) and Proterotheriidae (Pr) indicated. Toxodontian relationships are based on Shockey et al. [62], Bonini et al. [63], and Armella et al. [64]. Typothere relationships are based on Seoane and Cerdeño [65] and Croft and Anaya [66], with *Hemihegetotherium* sp. assumed to be closely related to *Hemihegetotherium achataleptum*. Typothere clades include Hegetotheriidae (He), Hegetotheriinae (H), Interatheriidae (Int), Mesotheriidae (Me), and Pachyrukhinae (P).

North American ungulate data (N = 73) are from Janis and Wilhelm [28]. Data for South American native ungulates (N = 36) are provided in S1 and S2 Tables. Regression lines are ordinary least-squared regressions.

Taxa include: *Thoatherium minusculum* (Proterotheriidae), YPPM-VPPU 15719 (reversed; from Scott 1910, pl. XIII, Fig 13 [78]); D*iadiaphorus majusculus* (Proterotheriidae), AMNH 9196 (modified from Scott 1910, pl. V, Fig 2 [78]); *Theosodon lydekkeri* (Macraucheniidae), AMNH 9269 (from Scott 1910, pl. XX, Fig 7 [78]); N*esodon imbricatus* (Toxodontidae), YPPM-VPPU 15968, (from Scott 1912, pl. XXV, Fig 9 [79]); *Scarrittia canquelensis* (Leontiniidae), AMNH 29585 (from Chaffee 1952, pl. 11, Fig 2 [80]); *Protypotherium australe* (Interatheriidae), AMNH 9149 (from Sinclair 1909, pl. V, Fig 1 [81]); *Miocochilius anomopodus* (Interatheriidae), UCMP 38091 (from Stirton 1953, pl. 27 [75]); *Eutypotherium lehmannnitchei* (Mesotheriidae), MLP 12–1701 (reversed); *Pachyrukhos moyani* (Hegetotheriidae), AMNH 9481 (reversed; from Sinclair 1909, pl. X, Fig 15 [81]). Scale bars equal 5 cm in upper row (litopterns, toxodonts) and 3 cm in lower row (typotheres).
