# Supplementary material for: No evidence for parallel evolution of cursorial limb adaptations among Neogene South American native ungulates (SANUs)
Source: PLoS One. 2021 Aug 17;16(8):e0256371. doi: 10.1371/journal.pone.0256371 (PMC8370646; doi:10.1371/journal.pone.0256371)

S1 Fig. Line drawing of a notoungulate right astragalus illustrating the dorsal astragalar foramen. The specimen (MLP 75-II-1-9) is from Loma Verde, Argentina, and pertains to a large isotemnid, perhaps *Thomashuxleya*. (A) dorsal view, anterior toward bottom; (B) posterior view, with inferior surface toward top of page. Abbreviations: af: superior astragalar foramen; at: astragalar trochlea; fs: flexor sulcus.

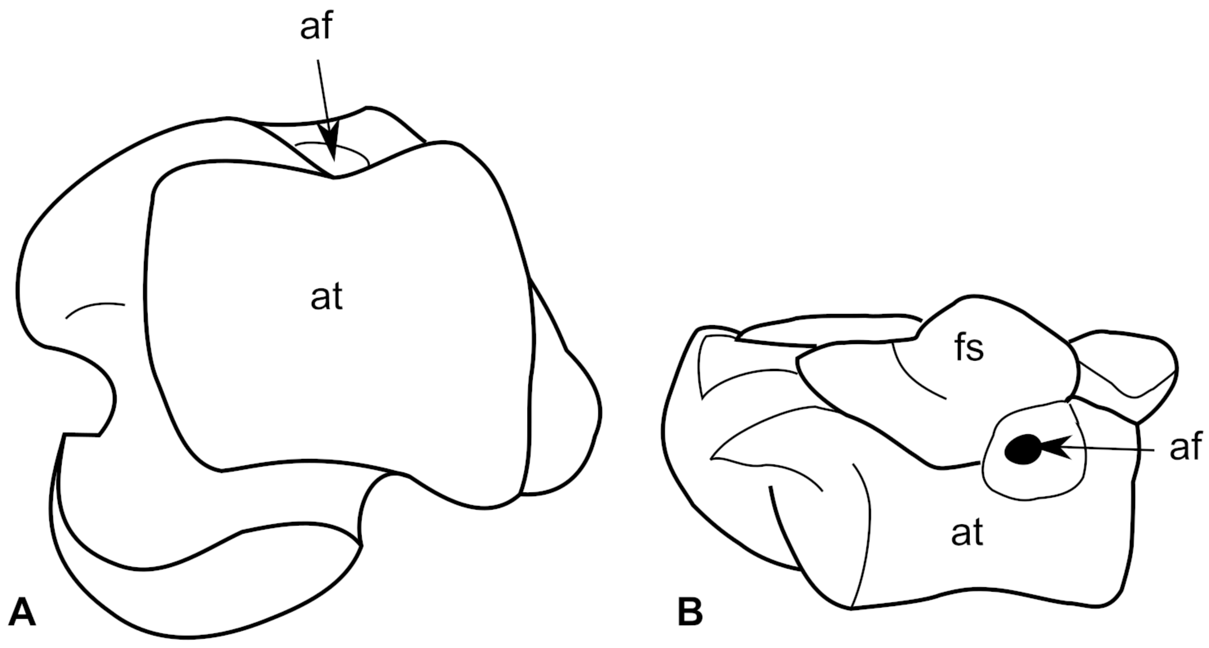

Supplement: S1 Fig — The specimen (MLP 75-II-1-9) is from Loma Verde, Argentina, and pertains to a large isotemnid, perhaps Thomashuxleya. (A) dorsal view, anterior toward bottom; (B) posterior view, with inferior surface toward top of page. Abbreviations: af: Superior astragalar foramen; at: Astragalar trochlea; fs: Flexor sulcus. (PDF) [file pone.0256371.s001.pdf]
