# Supplementary material for: No evidence for parallel evolution of cursorial limb adaptations among Neogene South American native ungulates (SANUs)
Source: PLoS One. 2021 Aug 17;16(8):e0256371. doi: 10.1371/journal.pone.0256371 (PMC8370646; doi:10.1371/journal.pone.0256371)
Supplement: S1 Table — Species are grouped by family within one of three larger clades: Litopterns (Litop), toxodont notoungulates (N:Tox), and typothere notoungulates (N:Typ). Institutional abbreviations: ACM, Beneski Museum of Natural History, Amherst College, USA; AMNH FM, fossil mammal collection, American Museum of Natural History, New York, USA; FMNH PM, Fossil Mammals collection, The Field Museum, Chicago, USA; IGM, Instituto de Geociencias y Minería, Bogotá, Colombia; MACN, Museo Argentino de Ciencias Naturales, Buenos Aires, Argentina; MCNAM-PV, vertebrate paleontology collections, Museo de Ciencias Naturales y Antropológicas “J. C. Moyano”, Mendoza, Argentina; MLP, Museo de La Plata, Argentina; MNHN-BOL-V, vertebrate paleontology collections, Museo Nacional de Historia Natural, La Paz, Bolivia; UATF-V, vertebrate paleontology collections, Universidad Autónoma “Tomás Frías”, Potosí, Bolivia; UCMP, University of California Museum of Paleontology, Berkeley, USA; UF, Florida Museum of Natural History, University of Florida, Gainesville, USA; YPM VPPU, Princeton University Collection, Yale Peabody Museum, New Haven, USA. (PDF) [file pone.0256371.s002.pdf]

S1 Table. Femur (F) and third metatarsal (Mt3) data for South American native ungulates analyzed in this study. Species are listed alphabetically by family within one of three larger clades: litopterns (Litop), toxodont notoungulates (N:Tox), and typothere notoungulates (N:Typ). The Source column indicates how measurement values were collected: specimen = from a museum specimen with calipers; photo = from a specimen photo using ImageJ [1]; a reference citation = taken directly from the literature. Institutional abbreviations: ACM, Beneski Museum of Natural History, Amherst College, USA; AMNH FM, fossil mammal collection, American Museum of Natural History, New York, USA; FMNH PM, Fossil Mammals collection, The Field Museum, Chicago, USA; IGM, Instituto de Geociencias y Minería, Bogotá, Colombia; MACN, Museo Argentino de Ciencias Naturales, Buenos Aires, Argentina; MCNAM-PV, vertebrate paleontology collections, Museo de Ciencias Naturales y Antropológicas "J. C. Moyano", Mendoza, Argentina; MLP, Museo de La Plata, Argentina; MNHN-BOL-V, vertebrate paleontology collections, Museo Nacional de Historia Natural, La Paz, Bolivia; UATF-V, vertebrate paleontology collections, Universidad Autónoma "Tomás Frías", Potosí, Bolivia; UCMP, University of California Museum of Paleontology, Berkeley, USA; UF, Florida Museum of Natural History, University of Florida, Gainesville, USA; YPM VPPU, Princeton University Collection, Yale Peabody Museum, New Haven, USA.

**Group**	**Family**	**Species**	**Femur (mm)**	**Mt3 (mm)**	**Mt3/F**	**Source**	**Specimen Number(s)**	**Comments**
Litop	Macraucheniidae	*Cramauchenia insolita*	247.0	105	0.43	Specimen	FMNH PM 13301	
Litop	Macraucheniidae	*Coniopternium andinus*	273.4	122	0.45	Photo	ACM 3287	ID follows Cifelli and Villarroel [2]. See also Loomis [3]
Litop	Macraucheniidae	*Llullataruca shockeyi*	243.2	129	0.53	Specimen	UATF-V-001904, UATF-V-001242	
Litop	Macraucheniidae	*Macrauchenia patachonica*	595	219	0.37	[4]	MACN 2	

Group	Family	Species	Femur (mm)	Mt3 (mm)	Mt3/F	Source	Specimen Number(s)	Comments
Litop	Macraucheniidae	*Theosodon garrettorum*	325	142	0.44	[5]	YPM VPPU 15164	
Litop	Proterotheriidae	*Anisolophus floweri*	192	82	0.43	[5]	YPM VPPU 15711	ID follows Soria [6]
Litop	Proterotheriidae	*Diadiaphorus majusculus*	224	92	0.41	[5]	YPM VPPU 15799	
Litop	Proterotheriidae	*Eoauchenia primitiva*	162.5	108.9	0.67	Specimen	MLP 48-XII-16-1, MLP 48-XII-16-7	ID follows Soria [6] and Shockey [7]
Litop	Proterotheriidae	*Megadolodus molariformis*	192	81.5	0.42	[8]	IGM 183544/183916	Juvenile
Litop	Proterotheriidae	*Protheosodon coniferus*	173	49	0.28	Specimen	ACM 3001	Courtesy of H. Singleton
Litop	Proterotheriidae	*Thoatherium minusculum*	141	83	0.59	[5]	YPM VPPU 15719	
N:Tox	Homalodotheriidae	*Homalodotherium cunninghami*	455.2	77	0.17	[9, 10]	FMNH PM 13092	
N:Tox	Leontiniidae	*Scarrittia canquelensis*			0.24	[11]	AMNH FM 29578	Calculated from indices (Femorotibial x Tibiometatarsal)
N:Tox	Leontiniidae	*Scarrittia canquelensis*			0.20	[11]	AMNH FM 29581	Calculated from indices (Femorotibial x Tibiometatarsal)
N:Tox	Notohippidae	*Eurygenium pacegnum*	175	44.4	0.25	[12]	MNHN-BOL-V-003644	Mean of L and R elements
N:Tox	Notohippidae	*Rhynchippus equinus*	202	74	0.37	[3]	ACM 3291	
N:Tox	Toxodontidae	*Adinotherium ovinum*	187	60	0.32	[13]	YPM VPPU 15131	
N:Tox	Toxodontidae	*Nesodon imbricatus*	373	75	0.20	[13, 14]	YPM VPPU 15968	
N:Tox	Toxodontidae	*Toxodon* sp.			0.17	Specimen	MLP 12-1126	
N:Typ	Hegetotheriidae	*Hegetotherium mirabile*	110	43	0.39	[15]	YPM VPPU 15431, YPM VPPU 15392	
N:Typ	Hegetotheriidae	*Hemihegetotherium* sp.	120	41.5	0.35	Specimen	FMNH PM 14413	Femur is missing small portion of distal end)

Group	Family	Species	Femur (mm)	Mt3 (mm)	Mt3/F	Source	Specimen Number(s)	Comments
N:Typ	Hegetotheriidae	*Hemihegetotherium trilobus*	145	52.5	0.36	Specimen	UATF-V-001368, UATF-V-001369, UATF-V-001591	Femur estimated based on overlapping fragments
N:Typ	Hegetotheriidae	*Hemihegetotherium torresi*	117.9	40.1	0.34	Specimen	MLP 27-VIII-1-1	
N:Typ	Hegetotheriidae	*Pachyrukhos moyani*	70	31	0.44	[15]	YPM VPPU 9242	
N:Typ	Hegetotheriidae	*Paedotherium* sp.	59	29	0.49	[16]	MACN 7520	
N:Typ	Hegetotheriidae	*Paedotherium insigne*	77	34.5	0.45	[16]	MACN-A 6436	ID follows Cerdeño and Bond [17]
N:Typ	Hegetotheriidae	*Propachyrucos ameghinorum*	85	33	0.39	[18]	AMNH FM 29574	Femur is estimated
N:Typ	Interatheriidae	*Federicoanaya sallaensis*	82.3	30.8	0.37	[19]	MNHN-BOL-V-004500	Left MT
N:Typ	Interatheriidae	*Federicoanaya sallaensis*	82.3	32	0.39	[19]	MNHN-BOL-V-004500	Right MT
N:Typ	Interatheriidae	*Interatherium extensum*	63	20.8	0.33	[15]	YPM VPPU 15041	
N:Typ	Interatheriidae	*Interatherium robustum*	59	21	0.36	[15]	YPM VPPU 15401	
N:Typ	Interatheriidae	*Miocochilius anomopodus*	120.3	43.9	0.36	[20]	UCMP 38091	
N:Typ	Interatheriidae	*Protypotherium attenuatum*	78.0	35	0.45	[15]	YPM VPPU 15341	
N:Typ	Interatheriidae	*Protypotherium australe*	97.0	40	0.41	[15]	YPM VPPU 9149	
N:Typ	Interatheriidae	*Protypotherium* sp.			0.41	Specimen	MLP 90-XII-26-10	
N:Typ	Mesotheriidae	*Eutypotherium lehmannnitchei*	167.1	45	0.20	[14], Photo	MLP 71-IX-2-9, MLP 12-1701	
N:Typ	Mesotheriidae	*Typotheriopsis internum*	230	77	0.33	Specimen	FMNH PM 14452	Femur is estimated (a small bit missing at distal end)
N:Typ	Mesotheriidae	*Mesotherium cristatum*	240	60	0.25	[21]	(unknown)	

Group	Family	Species	Femur (mm)	Mt3 (mm)	Mt3/F	Source	Specimen Number(s)	Comments
N:Typ	Mesotheriidae	*Trachytherus alloxus*	179	60	0.34	[22]	UF 90960	
N:Typ	Oldfieldthomasiidae	*Allalmeia atalaensis*	82	28	0.34	[23]	MCNAM-PV 507	

1. Schneider CA, Rasband WS, Eliceiri KW. NIH Image to ImageJ: 25 years of image analysis. Nature Methods. 2012;9: 671-5. doi: 10.1038/nmeth.2089.
2. Cifelli RL, Soria MF. Notes on Deseadan Macraucheniidae. Ameghiniana. 1983;20: 141-53.
3. Loomis FB. The Deseado Formation of Patagonia. Concord, New Hampshire: Runford Press; 1914.
4. Parodi LJ. Huesos de los miembros de los macroquénidos neoterciarios. Physis. 1931;10: 294-304.
5. Scott WB. Mammalia of the Santa Cruz Beds. Volume VII, Paleontology. Part I, Litopterna. In: Scott WB, editor. Reports of the Princeton University Expeditions to Patagonia, 1896-1899. Stuttgart: Princeton University, E. Schweizerbart'sche Verlagshandlung (E. Nägele); 1910. pp. 1-156.
6. Soria MF. Los Proterotheriidae (Mammalia, Litopterna), sistemática, origen y filogenia. Monogr Mus Argent Cienc Nat. 2001;1: 1-167.
7. Shockey BJ. Specialized knee joints in some extinct, endemic, South American herbivores. Acta Palaeontol Pol. 2001;46(2): 277-88.
8. Cifelli RL, Villarroel C. Paleobiology and affinities of *Megadolodus*. In: Kay RF, Madden RH, Cifelli RL, Flynn JJ, editors. Vertebrate Paleontology in the Neotropics: The Miocene Fauna of La Venta, Colombia. Washington, D.C.: Smithsonian Institution Press; 1997. pp. 265-88.
9. Elissamburu A. Estudio biomecánico y morfofuncional del esqueleto apendicular de *Homalodotherium* Flower 1873 (Mammalia, Notoungulata). Ameghiniana. 2010;47(1): 25-43.
10. Scott WB. A partial skeleton of *Homalodontotherium* from the Santa Cruz beds of Patagonia. Memoirs of the Field Museum of Natural History. 1930;50(1): 1-34.

11. Chaffee RG. The Deseadan vertebrate fauna of the Scarritt Pocket, Patagonia. Bull Am Mus Nat Hist. 1952;98: 509-62.
12. Shockey BJ. Two new notoungulates (Family Notohippidae) from the Salla Beds of Bolivia (Deseadan: late Oligocene): systematics and functional morphology. J Vertebr Paleontol. 1997;17(3): 584-99.
13. Scott WB. Mammalia of the Santa Cruz Beds. Volume VI, Paleontology. Part II, Toxodonta. In: Scott WB, editor. Reports of the Princeton University Expeditions to Patagonia, 1896-1899. Stuttgart: Princeton University, E. Schweizerbart'sche Verlagshandlung (E. Nägele); 1912. pp. 111-238.
14. Elissamburu A. Estimación de la masa corporal en géneros del Orden Notoungulata. Estud Geol (Madr). 2012;68(1): 91-111.
15. Sinclair WJ. Mammalia of the Santa Cruz Beds. Volume VI, Paleontology. Part I, Typotheria. In: Scott WB, editor. Reports of the Princeton University Expeditions to Patagonia, 1896-1899. Stuttgart: Princeton University, E. Schweizerbart'sche Verlagshandlung (E. Nägele); 1909. pp. 1-110.
16. Kraglievich L. Sobre el conducto humeral en las vizcachas y paquirucos chapadmalenses con descripción del *Paedotherium imperforatum*. Anales del Museo Nacional de Historia Natural "Bernardino Rivadavia". 1926;34: 45-88.
17. Cerdeño E, Bond M. Taxonomic revision and phylogeny of *Paedotherium* and *Tremacyllus* (Pachyrukhinae, Hegetotheriidae, Notoungulata) from the late Miocene to Pleistocene of Argentina. J Vertebr Paleontol. 1998;18(4): 799-811.
18. Simpson GG. A Deseado hegetothere from Patagonia. Am J Sci. 1945;243: 550-64.
19. Hitz RB, Billet G, Derryberry D. New interatheres (Mammalia, Notoungulata) from the late Oligocene Salla Beds of Bolivia. J Paleontol. 2008;82(3): 447-69.
20. Stirton RA. A new genus of interatheres from the Miocene of Colombia. Univ Calif Publ Geol Sci. 1953;29(6): 265-348.
21. Serres AERA. De l'ostéographie du *Mesotherium* et de ses affinitiés zoologiques. Comptes Rendus de la Académie des Sciences, Paris. 1867;65: 6-17, 140-8, 273-8, 429-37, 593-9, 740-8, 841-8.
22. Shockey BJ, Anaya F. Postcranial osteology of mammals from Salla, Bolivia (late Oligocene): form, function, and phylogenetic implications. In: Sargis EJ, Dagosto M, editors. Mammalian Evolutionary Morphology: A Tribute to Frederick S Szalay. New York: Springer; 2008. pp. 135-57.
23. Rusconi C. Algunos mamíferos, reptiles y aves del Oligoceno de Mendoza. Rev Soc Hist Geogr Cuyo. 1946;2: 1-37.
