# Supplementary material for: No evidence for parallel evolution of cursorial limb adaptations among Neogene South American native ungulates (SANUs)
Source: PLoS One. 2021 Aug 17;16(8):e0256371. doi: 10.1371/journal.pone.0256371 (PMC8370646; doi:10.1371/journal.pone.0256371)
Supplement: S2 Table — Ages (to nearest million years, my) are based on the provenance of the specimen(s) from which femur and metatarsal data were collected. The age listed is the value used for calculating regression equations and represents an approximate midpoint of the age for the formation or locality. Species are grouped by family within one of three larger clades: Litopterns (Litop), toxodont notoungulates (N:Tox), and typothere notoungulates (N:Typ). (PDF) [file pone.0256371.s003.pdf]

S2 Table. Approximate ages of South American native ungulates analyzed in this study. Ages (to nearest million years, my) are based on the provenance of the specimen(s) from which femur and metatarsal data were collected. The age listed is the value used for calculating regression equations and represents an approximate midpoint of the age for the formation or locality. Species are listed alphabetically by family within one of three larger clades: litopterns (Litop), toxodont notoungulates (N:Tox), and typothere notoungulates (N:Typ).

Group	Family	Species	Geological Group/Formation	Age (my)	Source(s)	Comments
Litop	Macraucheniidae	*Cramauchenia insolita*	Sarmiento (Colhuehuapian)	20	[1]	
Litop	Macraucheniidae	*Coniopternium andinus*	Sarmiento (Deseadan)	27	[1]	
Litop	Macraucheniidae	*Llullataruca shockeyi*	Honda Group (Bolivia)	13	[2]	
Litop	Macraucheniidae	*Macrauchenia patachonica*	Pampean "Formation"	0	[3]	
Litop	Macraucheniidae	*Theosodon garrettorum*	Santa Cruz Fm.	17	[4, 5]	Based on coastal outcrops
Litop	Proterotheriidae	*Anisolophus floweri*	Santa Cruz Fm.	17	[4, 5]	Based on coastal outcrops
Litop	Proterotheriidae	*Diadiaphorus majusculus*	Santa Cruz Fm.	17	[4, 5]	Based on coastal outcrops
Litop	Proterotheriidae	*Eoauchenia primitiva*	Monte Hermoso Fm.	5	[6]	
Litop	Proterotheriidae	*Megadolodus molariformis*	Honda Group (Colombia)	13	[7]	Based on Monkey Beds interval
Litop	Proterotheriidae	*Protheosodon coniferus*	Sarmiento Fm. (Deseadan)	27	[1]	
Litop	Proterotheriidae	*Thoatherium minusculum*	Santa Cruz Fm.	17	[4, 5]	Based on coastal outcrops
N:Tox	Homalodotheriidae	*Homalodotherium cunninghami*	Santa Cruz Fm.	17	[4, 5]	Based on coastal outcrops
N:Tox	Leontiniidae	*Scarrittia canquelensis*	Sarmiento Fm. (Deseadan)	27	[1]	

Group	Family	Species	Geological Group/Formation	Age (my)	Source(s)	Comments
N:Tox	Notohippidae	*Eurygenium pacegnum*	Salla Beds	26	[8]	
N:Tox	Notohippidae	*Rhynchippus equinus*	Sarmiento Fm. (Deseadan)	27	[1]	
N:Tox	Toxodontidae	*Adinotherium ovinum*	Santa Cruz Fm.	17	[4, 5]	Based on coastal outcrops
N:Tox	Toxodontidae	*Nesodon imbricatus*	Santa Cruz Fm.	17	[4, 5]	Based on coastal outcrops
N:Tox	Toxodontidae	*Toxodon* sp.	Pampean “Formation”	0	[3]	
N:Typ	Hegetotheriidae	*Hegetotherium mirabile*	Santa Cruz Fm.	17	[4, 5]	Based on coastal outcrops
N:Typ	Hegetotheriidae	*Hemihegetotherium* sp.	Corral Quemado Fm.	3	[9, 10]	
N:Typ	Hegetotheriidae	*Hemihegetotherium trilobus*	Honda Group (Bolivia)	13	[2]	
N:Typ	Hegetotheriidae	*Hemihegetotherium torresi*	Arroyo Chasicó Fm.	9	[11]	
N:Typ	Hegetotheriidae	*Pachyrukhos moyani*	Santa Cruz Fm.	17	[4, 5]	Based on coastal outcrops
N:Typ	Hegetotheriidae	*Paedotherium* sp.	“Hermosense”	5	[6, 12]	Based on age of Monte Hermoso
N:Typ	Hegetotheriidae	*Paedotherium insigne*	Chapadmalal Fm.	4	[6, 12]	
N:Typ	Hegetotheriidae	*Propachyrucos ameghinorum*	Sarmiento Fm. (Deseadan)	27	[1]	
N:Typ	Interatheriidae	*Federicoanaya sallaensis*	Salla Beds	26	[8]	
N:Typ	Interatheriidae	*Interatherium extensum*	Santa Cruz Fm.	17	[4, 5]	Based on coastal outcrops
N:Typ	Interatheriidae	*Interatherium robustum*	Santa Cruz Fm.	17	[4, 5]	Based on coastal outcrops
N:Typ	Interatheriidae	*Miocochilius anomopodus*	Honda Group (Colombia)	13	[7]	Based on Monkey Beds interval
N:Typ	Interatheriidae	*Protypotherium attenuatum*	Santa Cruz Fm.	17	[4, 5]	Based on coastal outcrops
N:Typ	Interatheriidae	*Protypotherium australe*	Santa Cruz Fm.	17	[4, 5]	Based on coastal outcrops

Group	Family	Species	Geological Group/Formation	Age (my)	Source(s)	Comments
N:Typ	Mesotheriidae	*Eutypotherium lehmannnitschei*	"Laguna Blanca"	12	[13, 14]	Based on Río Mayo Fm.
N:Typ	Mesotheriidae	*Typotheriopsis internum*	Andalhualá Fm.	7	[9, 10]	
N:Typ	Mesotheriidae	*Mesotherium cristatum*	Ensenada Formation	1	[15, 16]	
N:Typ	Mesotheriidae	*Trachytherus alloxus*	Salla Beds	26	[8]	

1. Dunn RE, Madden RH, Kohn MJ, Schmitz MD, Strömberg CAE, Carlini AA, et al. A new chronology for middle Eocene-early Miocene South American Land Mammal Ages. Geol Soc Am Bull. 2013;125(3-4): 539-55. doi: 10.1130/b30660.1.
2. MacFadden BJ, Anaya F, Perez H, Naeser CW, Zeitler PK, Campbell KE, Jr. Late Cenozoic paleomagnetism and chronology of Andean basins of Bolivia: evidence for possible oroclinal bending. J Geol. 1990;98: 541-55.
3. Fariña RA, Vizcaíno SF, Bargo MS. Body mass estimations in Lujanian (late Pleistocene - early Holocene of South America) mammal megafauna. Mastozool Neotrop. 1998;5(2): 87-108.
4. Fleagle JG, Perkins ME, Heizler MT, Nash B, Bown TM, Tauber AA, et al. Absolute and relative ages of fossil localities in the Santa Cruz and Pinturas formations. In: Vizcaíno SF, Kay RF, Bargo MS, editors. Early Miocene Paleobiology in Patagonia: High-Latitude Paleocommunities of the Santa Cruz Formation. Cambridge: Cambridge University Press; 2012. pp. 41-58.
5. Perkins ME, Fleagle JG, Heizler MT, Nash B, Bown TM, Tauber AA, et al. Tephnochronology of the Miocene Santa Cruz and Pinturas formations, Argentina. In: Vizcaíno SF, Kay RF, Bargo MS, editors. Early Miocene Paleobiology in Patagonia: High-Latitude Paleocommunities of the Santa Cruz Formation. Cambridge: Cambridge University Press; 2012. pp. 23-40.
6. Tomassini RL, Montalvo CI, Deschamps CM, Manera T. Biostratigraphy and biochronology of the Monte Hermoso Formation (early Pliocene) at its type locality, Buenos Aires Province, Argentina. J South Am Earth Sci. 2013;48: 31-42. doi: 10.1016/j.jsames.2013.08.002.

7. Kay RF, Madden RH. Mammals and rainfall: paleoecology of the middle Miocene at La Venta (Colombia, South America). J Hum Evol. 1997;32: 161-99.
8. Kay RF, MacFadden BJ, Madden RH, Sandeman H, Anaya F. Revised age of the Salla beds, Bolivia, and its bearing on the age of the Deseadan South American Land Mammal "Age". J Vertebr Paleontol. 1998;18(1): 189-99.
9. Marshall LG, Patterson B. Geology and geochronology of the mammal-bearing Tertiary of the Valle de Santa María and Río Corral Quemado, Catamarca Province, Argentina. Fieldiana Geol (NS). 1981;9: 1-80.
10. Reguero MA, Candela AM. Late Cenozoic mammals from the northwest of Argentina. In: Salfity JA, Marquillas RA, editors. Cenozoic Geology of the Central Andes of Argentina. Salta: SCS Publisher; 2011. pp. 411-26.
11. Zárate MA, Schultz PH, Blasi A, Heil C, King J, Hames W. Geology and geochronology of type Chasicoan (late Miocene) mammal-bearing deposits of Buenos Aires (Argentina). J South Am Earth Sci. 2007;23(1): 81-90.
12. Kraglievich L. Sobre el conducto humeral en las vizcachas y paquirucos chapadmalenses con descripción del *Paedotherium imperforatum*. Anales del Museo Nacional de Historia Natural "Bernardino Rivadavia". 1926;34: 45-88.
13. Vera B, González Ruiz L, Novo N, Martin G, Reato A, Tejedor MF. The Interatheriinae (Mammalia, Notoungulata) of the Friasian sensu stricto and Mayoan (middle to late Miocene), and the fossils from Cerro Zeballos, Patagonia, Argentina. J Syst Palaeontol. 2018: 1-21. doi: 10.1080/14772019.2018.1511387.
14. Kraglievich L. La Formación Friaseana del Río Frías, Río Fénix, Laguna Blanca, etc., y su fauna de mamíferos. Physis. 1930;10: 127-66.
15. Bond M. Quaternary native ungulates of southern South America. A synthesis. In: Rabassa J, Salemme M, editors. Quat South Am Antarct Penins. Rotterdam: A.A. Balkema; 1999. pp. 177-205.
16. Soibelzon E, Tonni EP, Bidegain JC. Cronología, magnetoestratigrafía y caracterización bioestratigráfica del Ensenadense (Pleistoceno Inferior-Medio) en la ciudad de Buenos Aires. Rev Asoc Geol Argent. 2008;63(3): 421-9.
