# Supplementary material for: No evidence for parallel evolution of cursorial limb adaptations among Neogene South American native ungulates (SANUs)
Source: PLoS One. 2021 Aug 17;16(8):e0256371. doi: 10.1371/journal.pone.0256371 (PMC8370646; doi:10.1371/journal.pone.0256371)
Supplement: S3 Table — Species are grouped by family within one of three larger clades: Litopterns (Litop), toxodont notoungulates (N:Tox), and typothere notoungulates (N:Typ). (PDF) [file pone.0256371.s004.pdf]

S3 Table. Estimated body masses (BM) of South American native ungulates analyzed in this study. Species are listed alphabetically by family within one of three larger clades: litopterns (Litop), toxodont notoungulates (N:Tox), and typothere notoungulates (N:Typ). BM estimates for taxa that lack a previously published BM were estimated based on close relatives, using femur length as a point of comparison (Table S1). In some cases (e.g., *Eoauchenia primitiva*), body mass was bracketed by a larger and a smaller species. In other cases (e.g., *Cramauchenia insolita*), BM was calculated assuming geometric similarity (e.g., a femur length of 10% greater was assumed to equate to a BM 33% greater), a technique that has previously been used by others to estimate BM in groups such as SANUs that lack closely similar extant relatives (e.g., [1-3]).

**Group**	**Family**	**Species**	**BM (kg)**	**Source**	**Comments on estimate**
Litop	Macraucheniidae	*Cramauchenia insolita*	55-75	-	Assumes geometric similarity with *Theosodon garrettorum*.
Litop	Macraucheniidae	*Coniopternium andinus*	74-100	-	Assumes geometric similarity with *Theosodon garrettorum*.
Litop	Macraucheniidae	*Llullataruca shockeyi*	35-55	[4]	
Litop	Macraucheniidae	*Macrauchenia patachonica*	1,100	[5]	
Litop	Macraucheniidae	*Theosodon garrettorum*	125-170	[4]	
Litop	Proterotheriidae	*Anisolophus floweri*	36	[6]	Citation is for *Anisolophus australis*.
Litop	Proterotheriidae	*Diadiaphorus majusculus*	82	[6]	
Litop	Proterotheriidae	*Eoauchenia primitiva*	20-30	-	Between *Anisolophus floweri* and *Thoatherium minusculum*.
Litop	Proterotheriidae	*Megadolodus molariformis*	40-60	[7]	
Litop	Proterotheriidae	*Protheosodon coniferus*	20-30	-	Between *Anisolophus floweri* and *Thoatherium minusculum*.

Group	Family	Species	BM (kg)	Source	Comments on estimate
Litop	Proterotheriidae	*Thoatherium minusculum*	15-28	[7]	
N:Tox	Homalodotheriidae	*Homalodotherium cunninghami*	200-400	[6, 8]	
N:Tox	Leontiniidae	*Scarrittia canquelensis*	280-370	[7]	Citation is for *Anayatherium fortis*.
N:Tox	Notohippidae	*Eurygenium pacegnum*	20	[9]	
N:Tox	Notohippidae	*Rhynchippus equinus*	30	-	Assumes geometric similarity with *Eurygenium pacegnum*.
N:Tox	Toxodontidae	*Adinotherium ovinum*	100	[6]	
N:Tox	Toxodontidae	*Nesodon imbricatus*	250-350	[7]	
N:Tox	Toxodontidae	*Toxodon* sp.	1,000-1,200	[7]	
N:Typ	Hegetotheriidae	*Hegetotherium mirabile*	5-8	[6, 10]	
N:Typ	Hegetotheriidae	*Hemihegetotherium* sp.	7-12	-	Between *Hegetotherium mirabile* and *Hemihegetotherium trilobus*.
N:Typ	Hegetotheriidae	*Hemihegetotherium trilobus*	9-17	[7]	
N:Typ	Hegetotheriidae	*Hemihegetotherium torresi*	7-12	-	Between *Hegetotherium mirabile* and *Hemihegetotherium trilobus*.
N:Typ	Hegetotheriidae	*Pachyrukhos moyani*	1-2	[6, 11]	
N:Typ	Hegetotheriidae	*Paedotherium* sp.	1-3	[12]	
N:Typ	Hegetotheriidae	*Paedotherium insigne*	1-3	[7]	Based on other species of *Paedotherium*.
N:Typ	Hegetotheriidae	*Propachyrucos ameghinorum*	1-4	-	Assumes geometric similarity with *Paedotherium insigne*.
N:Typ	Interatheriidae	*Federicoanaya sallaensis*	2-4		Similar to *Protypotherium attenuatum*.
N:Typ	Interatheriidae	*Interatherium extensum*	1-3	[7]	Similar to *Interatherium robustum*.
N:Typ	Interatheriidae	*Interatherium robustum*	1-3	[7]	

Group	Family	Species	BM (kg)	Source	Comments on estimate
N:Typ	Interatheriidae	*Miocochilius anomopodus*	11-13	[7]	
N:Typ	Interatheriidae	*Protypotherium attenuatum*	2-4	[6]	
N:Typ	Interatheriidae	*Protypotherium australe*	5-8	[6, 10]	
N:Typ	Mesotheriidae	*Eutypotherium lehmannnitschei*	19-23	-	Based on femur length (S1 Table), assuming geometric similarity with *Trachytherus alloxus*.
N:Typ	Mesotheriidae	*Typotheriopsis internum*	51-59	-	Based on femur length (S1 Table), assuming geometric similarity with *Trachytherus alloxus*.
N:Typ	Mesotheriidae	*Mesotherium cristatum*	58-67	-	Based on femur length (S1 Table), assuming geometric similarity with *Trachytherus alloxus*.
N:Typ	Mesotheriidae	*Trachytherus alloxus*	24-28	[7]	

1. Vizcaíno SF, Bargo MS, Cassini GH. Dental occlusal surface area in relation to body mass, food habits and other biological features in fossil xenarthrans. Ameghiniana. 2006;43(1): 11-26.
2. Cassini GH, Mendoza M, Vizcaíno SF, Bargo MS. Inferring habitat and feeding behaviour of early Miocene notoungulates from Patagonia. Lethaia. 2011;44(2): 153-65. doi: 10.1111/j.1502-3931.2010.00231.x.
3. Vizcaíno SF, Cassini GH, Toledo N, Bargo MS. On the evolution of large size in mammalian herbivores of Cenozoic faunas of southern South America. In: Patterson BD, Costa LP, editors. Bones, Clones, and Biomes The History and Geography of Recent Neotropical Mammals. Chicago: University of Chicago Press; 2012. pp. 76-101.
4. McGrath AJ, Anaya F, Croft DA. Two new macraucheniids (Mammalia: Litopterna) from the late middle Miocene (Laventan South American Land Mammal Age) of Quebrada Honda, Bolivia. J Vertebr Paleontol. 2018;38(3): e1461632. doi: 10.1080/02724634.2018.1461632.
5. Fariña RA, Blanco RE, Christiansen P. Swerving as the escape strategy of *Macrauchenia patachonica* Owen (Mammalia; Litopterna). Ameghiniana. 2005;42(4): 751-60.

6. Cassini GH, Cerdeño E, Villafañe AL, Muñoz NA. Paleobiology of Santacrucian native ungulates (Meridiungulata; Astrapotheria, Litopterna and Notoungulata). In: Vizcaíno SF, Kay RF, Bargo MS, editors. Early Miocene Paleobiology in Patagonia: High-Latitude Paleocommunities of the Santa Cruz Formation. Cambridge: Cambridge University Press; 2012. pp. 243-86.
7. Croft DA. Horned Armadillos and Rafting Monkeys: The Fascinating Fossil Mammals of South America. Bloomington, Indiana: Indiana University Press; 2016.
8. Croft DA, Gelfo JN, López GM. Splendid innovation: The South American native ungulates. Annu Rev Earth Planet Sci. 2020;48: 249-90. doi: 10.1146/annurev-earth-072619-060126.
9. Shockey BJ. Two new notoungulates (Family Notohippidae) from the Salla Beds of Bolivia (Deseadan: late Oligocene): systematics and functional morphology. J Vertebr Paleontol. 1997;17(3): 584-99.
10. Croft DA, Anaya F. A new typothere notoungulate (Mammalia: Interatheriidae), from the Miocene Nazareno Formation of southern Bolivia. Ameghiniana. 2020;52(2): 189-208. doi: 10.5710/AMGH.11.01.2020.3271.
11. Macrini TE, Flynn JJ, Ni X, Croft DA, Wyss AR. Comparative study of notoungulate (Placentalia, Mammalia) bony labyrinths and new phylogenetically informative inner ear characters. J Anat. 2013;223(5): 442-61. doi: 10.1111/joa.12108.
12. Elissamburu A. Análisis morfométrico y morfofunctional del esqueleto appendicular de *Paedotherium* (Mammalia, Notoungulata). Ameghiniana. 2004;41(3): 363-80.
