# Supplementary material for: No evidence for parallel evolution of cursorial limb adaptations among Neogene South American native ungulates (SANUs)
Source: PLoS One. 2021 Aug 17;16(8):e0256371. doi: 10.1371/journal.pone.0256371 (PMC8370646; doi:10.1371/journal.pone.0256371)
Supplement: S1 File — Note the independent increase in Mt:F in several families of ungulates from the Eocene to the early Miocene. From Janis and Wilhelm [17]. (PDF) [file pone.0256371.s005.pdf]

S1 File. Graph of metatarsal-femur ratio (Mt:F) in Eocene through Pleistocene North American ungulates and large carnivores (> 7 kg). Note the independent increase in Mt:F in several families of ungulates from the Eocene to the early Miocene. From Janis and Wilhelm [1].

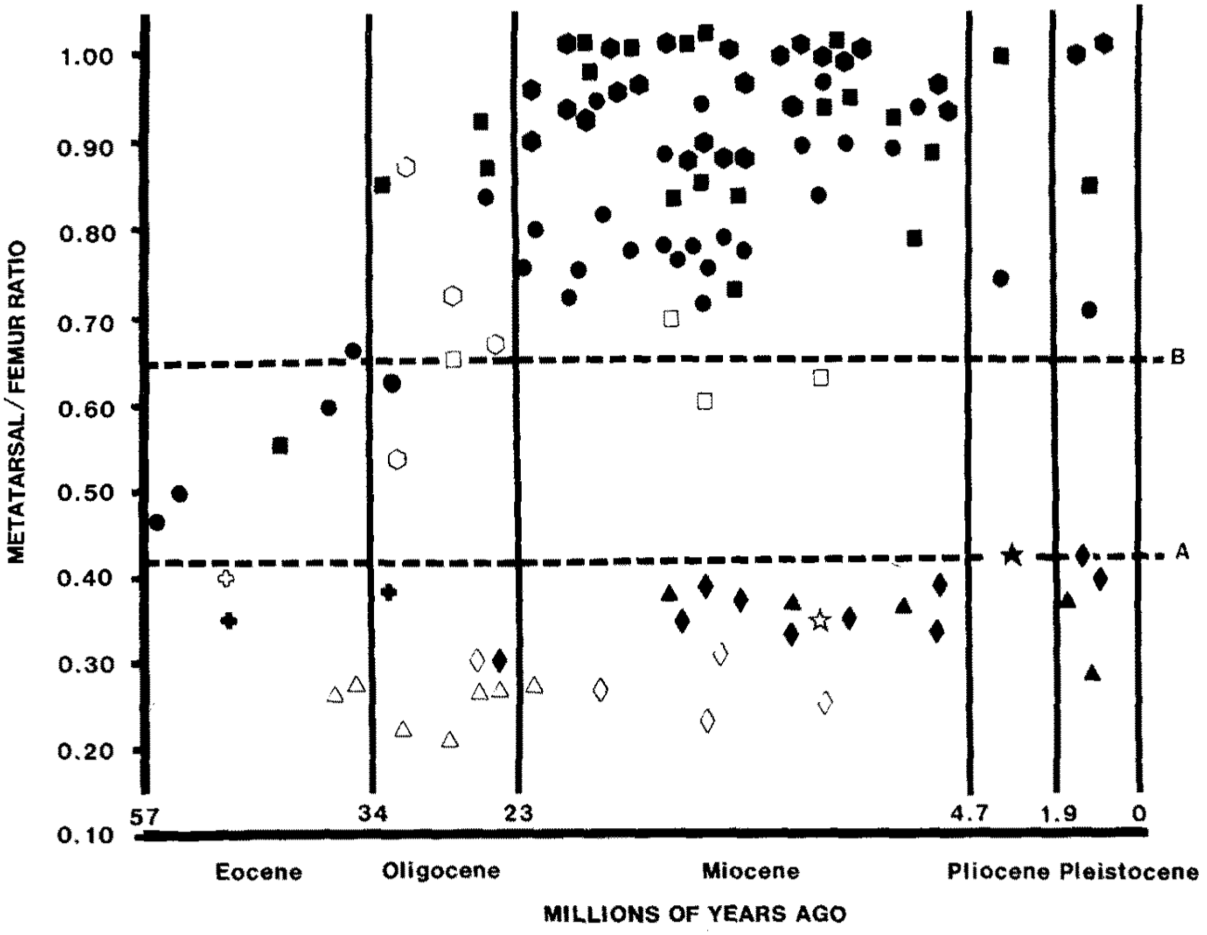

Fig. 1. Metatarsal-to-femur ratios in North American post-Paleocene Cenozoic (Tertiary plus Pleistocene) carnivores (of body mass greater than 7 kg) and ungulates. Open cross, mesonychid "condylarth"; filled crosses, hyaenodontid creodonts; open triangles, nimravids; filled triangles, felids; open diamonds, amphicyonids; filled diamonds, canids; open star, hemicyonine ursid (extinct subfamily of "dog bears"); filled star, hyenid; open squares, protoceratids; filled squares, camelids; open pentagons, traguloid ruminants; filled pentagons, pecoran ruminants; filled circles, equids. (A) Lower limit of MT/F ratio for present-day pursuit predators (note that all carnivorous taxa fall on or below this line and all herbivorous ungulate taxa fall above this line); (B) lower limit of MT/F ratio for present-day cursorial ungulates (camelids, pecoran ruminants, and equids). (Modified from Janis, 1994.)

1. Janis CM, Wilhelm PB. Were there mammalian pursuit predators in the Tertiary? Dances with wolf avatars. J Mamm Evol. 1993;1(2): 103-25.
