# Supplementary material for: No evidence for parallel evolution of cursorial limb adaptations among Neogene South American native ungulates (SANUs)
Source: PLoS One. 2021 Aug 17;16(8):e0256371. doi: 10.1371/journal.pone.0256371 (PMC8370646; doi:10.1371/journal.pone.0256371)
Supplement: Attachment — Submitted filename: Response to Reviewers Croft and Lorente.docx [file pone.0256371.s006.docx]

Below, our responses are in red so that they are easily distinguished from the reviewer comments.

Darin Croft and Malena Lorente

***********************************

Reviewer #1: This is a well-written and nicely composed manuscript that furthers our knowledge about mammalian anatomy and adaptations over the course of the Cenozoic. The approach is traditional, and there is no attempt at complex statistical analysis – rather the data are portrayed as a figure where the morphological trends over time in the different lineages can clearly be seen. This approach is entirely appropriate for the type of data obtained and the questions being addressed in this MS, and I do hope that the other reviewer(s) don’t insist on some sort of phylogenetic multivariate analysis which would serve to confabulate, rather than illuminate, the issues here.

The authors address the issue of the commonly used “index of cursoriality” of metatarsal to femur (Mt/F) ratio employed for some modern ungulate groups (primarily equids and ruminant + camelid artiodactyls) and whether it applies to the South American native ungulates (SANUs). These animals certainly appear to be short-legged in comparison with the modern groups, even if they parallel them in some other regards (e.g., loss of digits), and I’m delighted that the authors have actually done this study (for which obtaining data from South American museums was key). They document not only the relatively shorter legs of the SANUs, but also that there is no overall trend for increasing the ratio over time (again in contrast with the modern ungulate lineages, where in North America a ‘step-up’ increase has been documented, correlated with an opening of the habitat in the Oligocene). The conclusions notes that trends noted in modern ungulate lineages should not necessarily be assumed to apply to other groups, and that data from southern continents should be added to our studies in order to better understand evolutionary trends and trajectories.

The figures are clear, and the SI provides comprehensive information about the specimens studied and the original measurements.

I don’t really have any major issues with this MS, which I think sets out what it plans to do very well, and is a good contribution to our knowledge. However, I do think that it could benefit from a little more consideration about the issue of what the Mt/F ratio actually means, and its distribution among extant ungulates.

We appreciate the very positive evaluation and insightful suggestions of Dr. Janis!

Basically, a very high ratio (> 0.7) appears to be primarily a feature of equids (at least anchitheres and equines), and ruminant and camelid artiodactyls. I’d like to say that all of these taxa are unguligrade, but -------: secondarily digitigrade camelids don’t count, but most anchitheres have metatarsals as long (or longer than) modern Equus, and they supposedly were sub-unguligrade with a pad foot. Then you have suoids, which are unguligrade but have a low MT/F ratio, so maybe part of this relates to lateral digit reduction (although again here anchitheres upset the apple cart!). (A useful paper to consider here might be Clifford, JVP 2010, although unfortunately it only considers artiodactyls.) But lots of other ungulates (and paenungulates) from diversity of habitats (ceratomorphs, proboscideans, hyraxes) don’t have/never had (as far as we know for hyraxes) long metatarsals. So, a little more discussion about the diversity of today’s ungulates and their limb anatomy could be a good context for why one might expect, or not expect, SANUs to follow the so-called ‘established pattern’. Somewhere here you could also note that more cursorial carnivores also tend to have a higher ratio, even if they never get to be as high as the ungulates.

We have endeavored to incorporate this feedback as well as possible, given the structure of the manuscript. We have added discussions of modern mammal groups in several areas and integrated data from Clifford (2010) into the Litopterns section of the Discussion. We have added some comparisons with carnivores in the Notoungulates section of the Discussion.

Another thing to think about a bit more is what a high Mt/F ratio actually means in terms of function and biomechanics. You mention that it relates to the lengthening the distal limb relative to the proximal limb, but perhaps you could be a bit more explicit about relative femur length being relatively constant in ungulates and the “distal limb” usually meaning from the tibia on down. The phalanges are not usually lengthened (but see comment below). Longer limbs do give you longer strides, but in theory lengthening the femur could give you a longer limb (which is what humans and elephants both do). So what is the biomechanics of this anatomy, and why do things that way? This is probably getting into much more than is relevant to this MS, but there’s some stuff that Hildebrand has to say about this that could be added here.

We have expanded the “reasoning” for lengthening the distal limb in the introduction and cited some useful references (including Hildebrand 1985). We have also added a discussion of the phalanges and some other metrics for the third metatarsal to the Litopterns section of the Discussion.

What a longer limb does is give you a longer stride, which isn’t necessarily about speed (see comments below about correlation with habitat, etc.). There may be other reasons to what long limbs or a longer stride, or to *not* want them. If you’re a mediportal mammal, without an elastic energy spring ligament system of recovery in your limbs, it may not benefit you much at all, and then if you’re a hopper you lengthen the tibia more than the metatarsals. All too much for this particular MS, but a little bit of additional discussion could be useful!

We have tried to integrate this point into the text that we have added in several parts of the manuscript.

I also note that some litopterns, especially Thoatherium, have extremely long proximal phalanges, much more so than unguligrade ungulates (see brief discussion of this in Janis & Bernor, Frontiers in Ecology and Evolution, 2019). Is this somehow compensating for the shorter metapodials? What if one did a calculation of MT+PP/F ratio for all ungulates? (I’m not suggesting that you do that, but maybe mention that they appear to be doing something with their limb morphology quite unlike the equids they’re so often compared to.)

This is an interesting question that was also raised by Cifelli and Villarroel (1997) in their study of *Megadolodus*. We have collected additional measurements for *Thoatherium* and *Diadiaphorus*, compared them to published data for some other extant and extinct taxa, and added text discussing this question to the Litopterns section of the Discussion.

Some small issues:

p. 4: it would be nice to have a brief explanation of why the loss of the superior astragalar foramen was thought to be correlated with “cursoriality”.

Also, maybe rephrase a little (especially the final sentence in the paragraph): the correlation of anatomy with habitat is a secondary inference, the primary inference is the correlation of the anatomy with a particular type of locomotor performance (e.g., longer legs enable longer strides, but correlation with open habitat [or even speed] is a secondary inference).

We have expanded on the loss of the superior astragalar foramen and rephrased the final sentence of the paragraph. We have also added a new supplemental figure (S1 Fig) illustrating the foramen.

p. 4. Following on from the above, maybe mention that longer strides are actually correlated with locomotor efficiency at all gaits, although they may also enable greater speed. Also, Mt/F ratio declines over a more limited range of sizes than ‘several orders of magnitude’: as can be seen from Table II in Janis & Wilhelm 1993, a fox has a greater Mt/F ratio than a wolf, and a cat a greater one than a lion. (See also Fig 1 in Lovegrove & Mowoe, 2014).

We have noted that longer strides are more efficient and added citations to support that.

We have also modified the text to reflect that Mt:F can vary with body size on a smaller scale.

p. 5. Materials and methods. How were these measurements obtained? From the real specimens, by calipers? From the photos, from ImageJ? Also, to the uninitiated it may not be clear here what you mean by ‘endemic ungulate’ as previously you’ve been calling them SANUs.

This information was already included in S1 table, but we have added it to the manuscript under Materials and Methods and clarified it in the S1 table caption.

We also changed ‘endemic ungulate’ to SANU to avoid confusion.

p. 7. Although Mt/F ratios have only been documented for North American ungulates, note that similar ratios are seen in Old World ruminants and equids.

We have noted this and added citations.

p. 14. Line 3, should the reference be #35 rather than #6

Good catch! Original reference #6 (Lovegrove and Mowoe 2013) was changed to #35 (Lovegrove and Mowoe 2014).

p. 14. Note that the oreodonts were digitigrade, which was part of the reason that they were not included. Tayassuids and ceratomorphs were also not included, for similar reasons.

We have noted this in the text.

p. 14. 7 lines from the bottom ---- the only typo I’ve spotted so far! Should be “broad dietary preferences” (not “board”). Another minor error is in the SI: a couple of times you’ve put “Toxodon sp.” (sp. in italics) rather than “Toxodon sp.” (sp. in Roman type).

These typos have been corrected in the text, S1 Table, and S2 Table.

Fig. 1. At the expense of overloading the reader with information, perhaps there should be a little more information about who’s who in the North American ungulates (you mention on p. 12 that J&W note lower Mt/F ratios in the equids). I suggest identifying somehow the equids, the protoceratids + traguloids, and then the camelids + pecorans.

This is a good suggestion for how to include that information. We have used different colors to distinguish these three groups in the modified version of Figure 1 (new Figure 2) and have added the relevant information to the caption.

S3 Table. You say that body mass estimates are based on femur length. What is the reference group you are using to relate femur length to body mass. Do you have a reference for that?

This has been clarified in the caption, and several citations have been added.

Reviewer #2: Comments to the author
The spread of grasslands and the evolutionary response of ungulate mammals to the changing environments from closed to open landscapes is well-established in North America. Grasslands spread during the Neogene, and as a response ungulate mammals evolved teeth high higher crowns and longer limbs. However, it is unknown if the same evolutionary trends can be observed in other continents. South America is an excellent study case to evaluate if different evolutionary lineages of herbivorous mammals show similar adaptations to open environments. South America was isolated from other continents during most of the Cenozoic and a large diversity of endemic mammals evolved in relative isolation, including native ungulates (SANUs). This work is the first study to evaluate if SANUs showed the same pattern of limb elongation as North American ungulates in response to the spread of open landscapes. The finding that SANUs do not show a trend of limb elongation in response to the spread of open landscapes is somehow surprising although not completely unexpected, as SANUs have shown different patterns in the evolution of ecomorphological traits in comparison with North American ungulates (for example the early evolution of high-crown teeth in SANUs).

I consider that the manuscript is a valuable contribution to mammal palaeobiology, as it represents the first work to study patterns of limb elongation in South American ungulates across a broad geographical and temporal scales, providing the basis of comparison with evolutionary patterns seen in other continents. Therefore, I consider the work is suitable for publication, although I have several comments that I provide below and should be addressed first.

General comments
The authors discuss how the Mt:F values could be related with body size in some groups of SANUs. I think given these observations it would be appropriate to examine in more detail the possible allometric relationships between the Mt:F and body size.
Does any allometric pattern emerge for Notoungulates and/or Litopterns in a plot of log (Mt:F) vs log (body size)?

We examined this relationship as suggested by the reviewer and have integrated information about slopes and r-squared values of regression lines into the “Family-level patterns” section of the manuscript.

Title
I suggest avoiding the use of the acronym SANUs in the title.

This pronounceable acronym is increasingly common in the scientific literature and may be more immediately recognizable to some readers than South American native ungulates. Since it is included in addition to (rather than a substitute for) this longer term, we prefer to retain it in the title. However, if the editor agrees with the reviewer that it would be better to remove it, we can certainly do so.

Abstract
“None of the groups or subgroups we analyzed show a pronounced increase in Mt:F across this interval”
It would be helpful for the readers to clarify in the abstract if an increase in Mt:F is associated with limb elongation.

We changed “limb evolution” to “limb elongation’ in the preceding sentence to clarify that Mt:F is our proxy for limb elongation.

Introduction
• “Temperatures fluctuated but were relatively steady during the Oligocene and early Miocene and reached a peak around the early-middle Miocene, 18-16 million years ago”
Please add a reference for the dates of the Miocene Climatic Optimum. Notice that a recent review paper (Steinthorsdottir et al. 2021) on the Miocene climate uses dates of ca. 16.9 – 14.7 Ma for the climatic optimum; Steinthorsdottir, M., et al. (2021). The Miocene: The future of the past. Paleoceanography and Paleoclimatology, 36, e2020PA004037.

We had not seen this recent work, which is quite useful. The range has been adjusted and a citation added.

• “plant phytolith ecomorphology indicates that arid conditions and open habitats
were present in southern South America as early as the middle Eocene [23, 24].”
I consider is important to clarify (as discussed in the references cited by the authors) that the open habitats in southern South America during the Eocene were shrublands with abundant palms, a flora that was nonanalog to modern open savannas for example. Therefore, even though open habitats might have appeared in southern South America since the Eocene, the flora of these habitats was different from the open habitats that appeared in North America later during the Neogene.

Yes, this is important, and text has been added to clarify this point.

• “Despite longstanding interest in the so-called ‘precocious’ hypsodonty of South American notoungulates”
Here it would be relevant to cite Madden (2015), which in chapters 1 and 2 provides a detailed account on the issue of precocious hypsodonty in South American native herbivores, for the interested reader that may wish to know more about the ‘precocious’ hypsodonty in South America: Madden (2015). Hypsodonty in Mammals. Evolution, Geomorphology, and the Role of Earth Surface Processes. Cambridge University Press.

This has been added.

Materials and methods
• Please consider adding a figure of a cladogram showing the hypothesis of phylogenetic relationships of the genera of South American native ungulates sampled, showing the subclades that were analysed separately.

This is a helpful suggestion, and we have added a new figure showing these relationships (new Figure 1). We have also added a sentence in the introduction noting that molecular data suggest that the closest living relatives of these groups are perissodactyls.

• Please clarify if the least square regressions are Ordinary least square (OLS) or generalized least squares (GLS). Given that closely related species might tend to have more similar Mt:F values, the residuals might be correlated and in that case it would be preferable to use GLS over OLS.

We have clarified this in the text.

Discussion
- “As noted previously, North American ungulates show a marked increase in Mt:F
around the Oligocene-Miocene transition [14].”
Please see the comment below for Fig. 1. Although the trend of increase in Mt:F is reported in the literature, this trend is not clearly visible in the Fig. 1 as shown by the authors. Would a regression (as done for South American taxa) show this trend?

This increase is not evident in Figure 1 (new Figure 2) because it precedes the time range portrayed in that figure, which is based on the data available for SANUs. Although the figure could be modified to include the Eocene and Oligocene, this would compress the new SANU data presented in this work by 50%, making it more difficult to read. Instead, we have added a new supporting information file (S1 File) with the original graph of Janis and Wilhelm (1993) that illustrates this phenomenon. This new figure is cited in the Introduction, where this is first mentioned.

• “The taxa studied derive from a variety of habitats, nearly all of which have been reconstructed as at least partially open and suitable for more cursorial species.”
Please see the comments for Fig. 1, I think the information of habitat (open vs closed) could be indicated by the shape of the points, while the colours correspond to the subclades.

The SANUs are already distinguished by shapes as well as colors so that different groups can clearly be distinguished even if printed in black and white. We think trying to add additional information about the different habitats in which these animals lived would make the graph too difficult to interpret. In addition, we are not certain that this would even be possible since some taxa are from sites where the paleoenvironment has yet to be investigated.

• In addition to the relation of the femur and metatarsal III, the gear ratio of the calcaneus has been also used as a proxy of locomotor habit and posture in carnivorans (e.g. Polly 2010; Polly and Head, 2015). Although the gear ratio has not been studied in detail in ungulates, it could be a relevant metric to explore in future studies. It would be interesting to assess if the gear ratio shows similar patterns than the Mt:F in ungulates from North America and the SANUs.
Polly. 2010. Tiptoeing through the trophics: Geographic variation in carnivoran locomotor ecomorphology in relation to environment. In A. Goswami and A. Friscia (eds.), Carnivoran Evolution: New Views on Phylogeny, Form, and Function

Polly and Head. 2015. MEASURING EARTH-LIFE TRANSITIONS: ECOMETRIC ANALYSIS OF FUNCTIONAL TRAITS FROM LATE CENOZOIC VERTEBRATES. In: Earth-Life Transitions: Paleobiology in the Context of Earth System Evolution. The Paleontological Society Papers, Volume 21,

These works cannot be easily be extrapolated to Notoungulata and Litopterna because of differences in calcaneal morphology, but it would be very interesting to try this approach in the future.

Figure 1.
The data of Mt:F for North American ungulates showed in Fig. 1 clearly shows that these taxa had a higher Mt:F than South American native ungulates. However, it is not evident that there was an increase in the Mt:F values from the Oligocene to the Miocene. Would a regression line show this trend? (It does not seem it would). Is it necessary to add data from Paleogene North American ungulates to show clearly an increase in Mt:F since the Miocene? Please clarify.

This is addressed above under Discussion.

Since the colours of the points and lines differentiate the clades of SANUs (namely litopterns, toxodont notoungulates and typothere notoungulates), the information of whether a taxon has been hypothesized to have lived in a closed or open environment could be indicated by the shape of the points, for example squares and circles).

This is addressed above under Discussion.

Figure 2.
Please add a scale to the figure.

Scale bars have been added to this figure.

Supplementary Information
Table S1
Please explain how the Mt:F value were obtained for the specimens of Scarritia canquelensis that have no measurements of femur and Mt3 and were “computed from indices” as indicated in the comments.

This has been clarified in Table S1. Chaffee (1952) provides two ratios that can be multiplied to provide our ratio.
